# Effects of *Bacillus subtilis* Natto NB205 and Its Mutant NBMK308 on Egg Quality in Aging Laying Hens

**DOI:** 10.3390/life13051109

**Published:** 2023-04-29

**Authors:** Chaoyong Liao, Jian Cui, Jiaqi Lei, Yuming Guo, Bingkun Zhang

**Affiliations:** State Key Laboratory of Animal Nutrition, Department of Animal Nutrition and Feed Science, College of Animal Science and Technology, China Agricultural University, Beijing 100193, China

**Keywords:** aging laying hens, apoptosis-related genes, *B. subtilis* natto, magnum, pro-inflammatory cytokines, TJ proteins

## Abstract

In aging laying hens, reproductive changes reduce egg quality. *Bacillus subtilis* natto (*B. subtilis*) is a versatile bacterium with high vitamin K2 content, providing health benefits for animals and humans. This study investigated the effect of *B. subtilis* natto NB205 and its mutant NBMK308 on egg quality in aging laying hens. Results showed that NB205 and NBMK308 supplementation significantly improved albumen height (*p* < 0.001), Haugh units (*p* < 0.05), and eggshell thickness (*p* < 0.001) compared to the control group. Supplementation also increased ovalbumin expression, regulated tight junction (TJ) proteins, reduced pro-inflammatory cytokine levels, and improved the health and productivity of aging laying hens by regulating key apoptosis-related genes in the magnum part of the oviduct. There were differences in the expression of vitamin K-dependent proteins (VKDPs) in the magnum between NB205 and NBMK308, but no significant differences in the improvement of egg quality. Supplementation with NB205 and NBMK308 can improve egg quality in aging laying hens.

## 1. Introduction

In the poultry industry, egg quality is a critical issue that is influenced by several factors such as genetics, nutrition, environment, and age. Age, in particular, is a significant factor affecting egg quality in laying hens. Age has a significant effect on egg weight, shell thickness, and shell strength in the first laying cycle of White Leghorn strains [1]. The genome-wide analysis of lncRNA and mRNA expression in the uterus of laying hens during aging revealed molecular changes that may affect egg quality [2]. DNA methylome and transcriptome analysis identify key genes and pathways involved in speckled eggshell formation in aging laying hens [3]. Furthermore, chicken hypothalamic and ovarian DNA methylome alterations in response to forced molting may also contribute to changes in egg quality [4]. These findings emphasize that aging in laying hens leads to various physiological changes, including alterations in gene expression, DNA methylation, and hormonal balance, which ultimately result in a decline in egg quality.

Several studies are investigating the effects of dietary supplements on production and egg quality in aging laying hens. Recent research has highlighted additional nutritional interventions that may help to mitigate age-related declines in egg quality. Shudi Erzi San, a traditional Chinese herbal medicine, alleviates ovarian aging in laying hens [5]. The combination of quercetin and vitamin E improves egg production, egg quality, and immunity in aging breeder hens [6]. Se-enriched Cardamine violifolia improves laying performance and regulates ovarian antioxidant function in aging hens [7], while turmeric powder supplementation enhances production performance, blood biochemical parameters, and egg quality characteristics in laying hens [8]. The potential benefits of melatonin interventions and microbiota involvement in counteracting the effects of oxidative stress on the chicken ovary are demonstrated [7]. In another study, selenium yeast supplementation improves egg quality, plasma antioxidant levels, selenium deposition, and eggshell formation in aging laying hens [9]. Dietary isoflavone aglycons from soy germ pasta improve the reproductive performance of aging hens and lower cholesterol levels in egg yolks [10]. Although nutritional programs aim to improve egg quality in aging laying hens, their effectiveness is limited, and alternative methods must be found to address this issue. Further research is needed to determine the long-term effects of these methods on the health and productivity of aging laying hens.

Natto is a food that has been consumed in Japan for centuries and is made from fermented soybeans using *Bacillus subtilis* (*B. subtilis*) var. natto bacteria. *B. subtilis* natto is shown to improve animal production and meat quality, with studies indicating that *B. subtilis* natto N21 in combination with lactic acid bacteria enhances broiler growth performance, feed conversion ratio, and nutrient digestibility [11]. Cows fed *B. subtilis* natto exhibit increased milk production, improved rumen fermentation, and a positive effect on the ruminal microbiome [12], while *B. subtilis* natto also promotes rumen fermentation by modulating rumen microbiota in vitro [13]. In TOPIGS pigs, *B. subtilis* natto supplementation resulted in a decrease in skatole content and an increase in meat tenderness [14]. *B. subtilis* natto in two-stage fermented feather meal-soybean meal product (TSFP) enhances growth performance and immunity in finishing pigs [15]. *B. subtilis* natto has the potential to naturally improve livestock production by promoting the growth of beneficial bacteria and reducing harmful bacteria. *B. subtilis* natto is rich in vitamin K2 (VK2), which confers beneficial effects in osteoporosis [16,17], cardiovascular calcification [18], cognitive disease [19], inflammation [20], and diabetes [21]. Studies have shown that vitamin K-dependent proteins (VKDPs) play a critical role in bone health and aging in laying hens [22], with dietary interventions with VK and calcium improving bone and eggshell quality [22]. The longer half-life and potential health benefits of menaquinone-7 (MK-7), a form of VK2, have made it an attractive option for bone health [23]. Finally, mutagenesis breeding is a technique used to generate genetic variations in bacteria to develop new strains with desirable traits [24], which could lead to the production of new strains of *B. subtilis* natto with even more significant benefits for animal and human health.

In our lab, a strain of *B. subtilis* natto NB205 (NB205) was isolated from natto and was found to produce MK-7. Further mutation breeding led to the development of a mutant strain NBMK308, which had a much higher MK-7 titer. This discovery could have significant implications for industrial production of MK-7 and its potential applications. Therefore, we investigated the effect of NB205 and its mutant NBMK308 on the oviduct of aging laying hens in this study, as there is a lack of research on this topic. The results of this study provided valuable insights into the potential benefits of using *B. subtilis* natto in poultry production, which could improve egg quality, and confirmed the safety of using mutagenic bacteria. Thus, *B. subtilis* natto could be a promising probiotic candidate for improving the health and productivity of aging laying hens. However, further research is needed to investigate the underlying mechanisms of these effects and to optimize dosage and feeding strategies. In summary, this study contributed to our understanding of the potential benefits of using *B. subtilis* natto for improving egg quality in aging laying hens and provides a basis for future research in this area.

## 2. Materials and Methods

### 2.1. Preparation of Bacterial Powder

All cultures were grown aerobically with shaking at 180 rpm and 37 °C. Unless otherwise stated. Monoclonal NB205 and NBMK308 were cultured in 10 mL test tubes supplemented with 4 mL LB liquid medium for 12 h, then 400 μL cell solution was inoculated into 50 L flasks containing 10 L fermentation culture media for 36 h. Fermentation culture media: 6% glucose, 5% soy peptone, 1.5% yeast extract, 0.3% NaCl. The cell solutions were centrifuged at 5000 rpm for 10 min at 4 °C and the supernatant was discarded. A bacterial slurry containing 5% glycerol as a protectant was added at a ratio of 1:3.5 (g/mL) to prepare a bacterial suspension. Corn starch was used as a drying carrier, and the carrier and bacterial suspension were mixed in a ratio of 1:5 (g/mL) and then dried at 50 °C with air blast to prepare bacterial powder.

### 2.2. Birds, Diets, and Management

A total of one hundred and eighty 81-week-old Hy-Line brown laying hens in good physical condition with no difference in egg quality were fed a basal diet for 2 weeks and then randomly divided into 3 groups (6 replicates/group, 10 birds/replicate). The experimental treatments were (1) control group (treated with corn starch); (2) bacterial powder of NB205 group (108 CFU/kg); (3) bacterial powder of mutant NBMK308 (108 CFU/kg). The composition and nutrient levels of the basal diet are shown in Table 1 throughout the experiment. Layers (2 birds per cage) in each replicate were housed in 5 adjacent cages. An enclosed, ventilated, conventional room was maintained at 25 °C with a daily lighting schedule of 16 h light and 8 h dark. Food and water were provided ad libitum throughout the 8-week study.

### 2.3. Sample Collection

Hens were humanely sacrificed by electrocution for sampling. After slaughter, the magnum part of oviduct was quickly removed and rinsed with sterile saline. Part of each section (1 cm) was isolated, fixed in 4% paraformaldehyde, and stored at 4 °C for microscopic evaluation of morphology. Another part of each isolated section (1 cm) was collected and placed in liquid nitrogen for analysis.

#### Production Performance and Egg Quality

The production performance of laying hens was measured from 81 to 88 weeks of age. Daily egg production and egg weight were recorded per replicate unit. Abnormal eggs, including soft-shelled, cracked, and broken eggs, were also recorded daily. Feed intake was measured weekly. Feed conversion ratio (FCR, feed intake/egg weight) was calculated from egg production and feed intake. At 3-week intervals and at the end of the experiment, eggs from each treatment were selected for quality analysis. Egg weight, yolk color (Roche colorimetric method), Haugh unit, and albumen height were tested using a digital egg tester (EA-01, Orka Food Technology Ltd., Tel Aviv, Israel). Eggshell strength was tested using an Egg Force Reader (ESTG-01, Orka Food Technology Ltd, Tel Aviv, Israel). Eggshell thickness was measured at the large end, equatorial region, and small end using an eggshell thickness gauge. (EFR-01, Orka Food Technology Ltd, Tel Aviv, Israel).

### 2.4. Determination of the Number of Bacillus spp. in the Feed

Following the coning and quartering method, the feed was mixed with normal saline and diluted at 95 °C for 30 min, the *Bacillus* spp. coating plate was sieved and counted. The strain morphology was found to be consistent with the added *B. subtilis*, which was an order of magnitude higher than the expected amount added [25].

#### Determination of *Bacillus* spp. Counts in Feces

The 0.2 g of feces was placed in a centrifuge tube containing 20 mL of sterile normal saline and then bathed in a 95 °C water bath for 30 min. It was then vortexed and mixed. Sterile normal saline was diluted 10, 100, 1000 times. An amount of 100 μL of each dilution gradient solution was dropped onto the nutrient agar medium, and the bacteria solution was evenly spread on the medium using a bacteria stick. The bacterial solution was incubated at 37 °C for 24 h in an incubator, and the number of individual colonies was calculated. *B. subtilis* content in each sample (CFU) = dilution times × 100 × 10 × number of individual colonies.

### 2.5. Quantitative Real-Time PCR (qRT-PCR)

Magnum samples were harvested for total RNA extraction using TRIzol solution. RNA concentration was measured using a NanoDrop instrument (Thermo Fisher Scientific, Waltham, MA, USA) at 260 nm. The total RNA was then reverse transcribed into complementary DNA (cDNA) using StarScript II First-strand cDNA Synthesis Mix with Guide DNA (gDNA) Remover (GenStar, Shanghai, China). qRT-PCR was performed to determine the relative mRNA expression of target genes to the internal reference gene *β-actin* using 2X RealStar Green Fast Mixture (GenStar, Shanghai, China). The value of the quantification cycle (Cq), defined as the cycle in which the reporter fluorescence is distinguishable from the background in the extension phase of the PCR reaction, was averaged in sextuplicate. Quantified by real-time PCR using *β-actin* as a reference gene. The target gene primer sequences are shown in Supplementary Material Table S1.

### 2.6. Western Blotting

Western blotting for differentially expressed proteins was performed according to a previously reported method [26,27]. Frozen tissue extracts were prepared using RIPA buffer. Lysates were centrifuged to remove insoluble material. Proteins were separated by 6–12% SDS-PAGE gel; separated proteins were transferred to PVDF membranes (IPVH00010, Millipore, MA, USA) and probed with the following primary antibodies: ovalbumin antibody (ab306591, Abcam, Shanghai, China), β-actin antibody (T40104F, Abmart, Shanghai, China), interferon gamma (INF-γ) antibody (T56671S, Abmart, Shanghai, China), phospho-STAT3 (p-STAT3) (Y705) antibody (T40061, Abmart, Shanghai, China), STAT3 antibody (9139T, Cell Signaling Technology, Shanghai, China), and caspase-3 antibody (T40044S, Abmart, Shanghai, China). For each protein, one blot from four independent experiments was presented. The relative levels of protein expression were calculated from densitometric scans using ImageJ software and normalized to β-actin levels from four independent experiments.

### 2.7. Extraction of MK-7 from Jejunal Chyme and HPLC Analysis

For the extraction and analysis of MK-7, 0.1 g of jejunum chyme was taken and mixed with isopropanol and hexane in the ratio of 3/4/8 (fermentation broth/isopropanol/hexane, *Q*/*V*/*V*). For the extraction and analysis of MK-7, 0.1 g of fermentation broth jejunal chyme was taken and mixed with isopropanol and hexane in a ratio of 3/4/8 (fermentation broth/isopropanol/hexane, *Q*/*V*/*V*). The mixture was vortexed for 2 min to obtain a homogeneous suspension, then centrifuged at 3000 rpm for 10 min, the organic layer was collected for vacuum evaporation, and then the dried precipitate was redissolved in 100 µL of isopropanol, and the content of MK-7 in the solution was determined. The standard solution was prepared by accurately weighing 10.00 mg of MK-7 standard and dissolving in 20 mL of isopropanol for 10 min in a 50 °C water bath. The Agilent Infinity II Prime LC system coupled with a DAD detector (wavelength 254 nm) was used to analyze the MK-7 titer. Metabolite separation was achieved using a C18 column (Eclipse PlusC18 RRHD 1.8 μm, 2.1 × 50 mm). The mobile phase was acetonitrile: ethanol (50:50, *v*/*v*). A flow rate of 0.2 mL/min was used at a constant temperature of 40 °C in the column oven. The calibration curve was determined using five standard concentrations between 5 mg/L and 500 mg/L (Supporting Figure S1, linear regression).

### 2.8. Magnum Morphology Analysis

The magnums of oviduct specimens were first washed under running water, then dehydrated in different concentrations of ethanol, then immersed in xylene, then immersed in paraffin and embedded, then sectioned at 5 mm thickness, and finally stained with hematoxylin and eosin (Solarbio Science & Technology Co., Ltd., Beijing, China) for conventional morphological assessment. Images of the different parts of the oviduct were taken using a DM3000 microscope (Leica, Wetzlar, Germany). The length of the primary fold and the length of the secondary fold in the magnum were measured for 2 to 3 adjacent fold spaces in each section. Magnum morphology measurements were made using Image-Pro software (Media Cybernetics, Rockville, MD, USA).

### 2.9. Statistical Analysis

The data are presented as mean ± SEM (standard error). For statistical analysis, one-way ANOVA was performed, followed by Duncan’s multiple comparison test using SPSS version 20.0 (Chicago, IL, USA). The significance levels were indicated by asterisks as follows: * for 0.05 > *p*-value > 0.01, ** for 0.01 > *p*-value > 0.001, and *** for 0.001 > *p*-value.

## 3. Results

### 3.1. Fermentation of NB205 and NBMK308 for the Production of MK-7 and Evaluation of Its Efficacy as a Feed Additive for Aging Laying Hens

**Figure 1:**
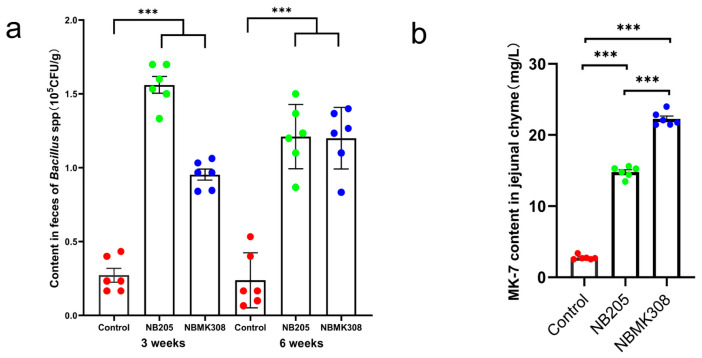
Efficacy test of supplementing diets with NB205 and NBMK308 for feeding aging laying hens. (**a**). The NB205 and NBMK308 groups of aging laying hens had consistently higher levels of *Bacillus* spp. spores in their feces than the control group in both the third and sixth weeks of the experiment. (**b**). Both NB205 and NBMK308 groups had significantly higher levels of MK-7 in their jejunal chyme compared to the control group, with the NBMK308 group having significantly higher levels than the NB205 group. Experiments were performed in sextuplicate (*n* = 6). *** for 0.001 > *p*-value.

NB205, capable of producing MK-7, was isolated from natto. Its MK-7 titer was determined to be 16.31 ± 2.234 mg/L. A mutant strain, NBMK308, was obtained from NB205 by mutation breeding, with a MK-7 titer of 35.43 ± 1.433 mg/L. This represented a 117.23% increase over the original NB205 and a 722.04% increase over *B. subtilis* 168 ([28]). After coning and quartering, the bacterial viability of the NB205 and NBMK308 bacterial powders was determined to be 1.8 × 10^10^ and 3.1 × 10^10^ CFU/Kg, respectively. Subsequently, after the powders were added to the diet, the levels of *B. subtilis* in the NB205 and NBMK308 groups were found to be 3.06 × 10^8^ and 2.00 × 10^8^ CFU/Kg, respectively. In the third and sixth week of the experiment, the number of *Bacillus.* spp. in the feces of the laying hens was measured and it was found that the spore content of the experimental group was significantly higher than that of the control group in the third week. Furthermore, it was observed that this difference remained stable until week six (Figure 1a). The content of MK-7 in the jejunal chyle of the NBMK308 group was significantly higher than that of the NB205 group, with both experimental groups showing significantly higher levels than the control group (Figure 1b, [28]).

*B. subtilis* has been shown to be capable of colonizing the intestinal tract. Studies have shown that *B. subtilis* can survive passage through the gastrointestinal tract and colonize the intestines of both humans and animals [29,30], where it can provide various health benefits such as modulating the immune system and producing antimicrobial compounds [31]. These results indicate that the high-yield MK-7 producing NB205 and NBMK308 were able to successfully colonize the intestinal tract of aging laying hens. Despite the differences between the intestinal environment and the conditions of in vitro fermentation, the strains were still able to produce MK-7 stably in the intestinal environment.

### 3.2. Production Performance and Egg Quality

The effects of dietary supplementation with the NB205 and NBMK308 groups on the performance of aging laying hens were shown in Table 2. It was observed that there were no significant differences in production performance between the treatments during the experimental period.

The effects of dietary supplementation with the NB205 and NBMK308 groups on egg quality in aging laying hens are shown in Table 3. Before the experiment, no significant differences were observed in eggshell parameters (thickness and strength), albumen weight, yolk color, and Haugh unit. However, in the third week of the experiment, the NBMK308 group showed significantly lower albumen height than the control group (*p* < 0.05), while the NBMK308 group showed significantly higher eggshell thickness than the control group (*p* < 0.001). The NB205 group had a significantly higher yolk color than the control group (*p* < 0.05). By the sixth week of the experiment, both the NB205 and NBMK308 groups showed significantly higher albumen height than the control group (*p* < 0.001). The NB205 and NBMK308 groups had significantly higher Haugh units than the control group (*p* < 0.05), and eggshell thickness was significantly higher in the NB205 and NBMK308 groups than in the control group (*p* < 0.001).

### 3.3. Expression of Ovalbumin and Morphological Observation in the Magnum of the Oviduct

**Figure 2:**
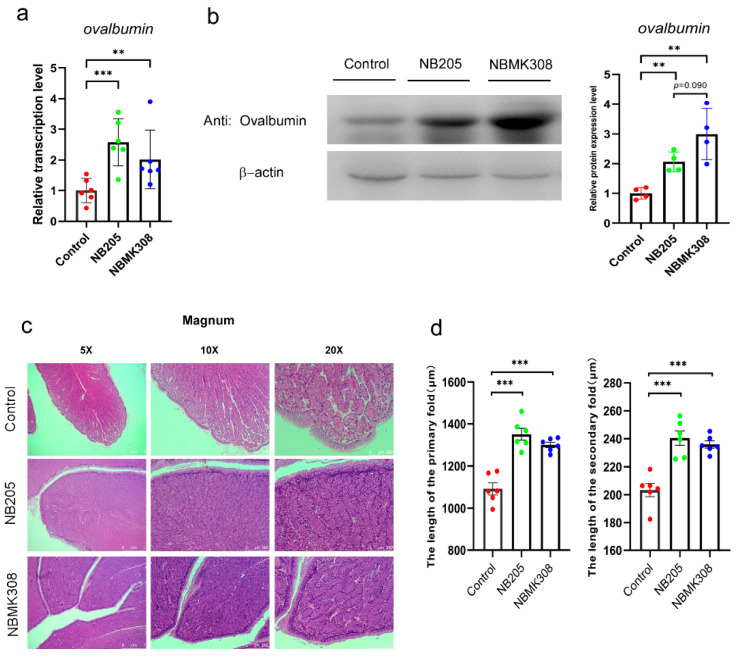
Expression of ovalbumin and morphological observation in the magnum. (**a**). Ovalbumin mRNA expression and (**b**). protein expression in the magnum. (**c**). Magnum was observed after H&E staining and microscopic observation. (**d**). Significant increase in primary and secondary fold length in the NB205 and NBMK308 groups. Experiments were performed in sextuplicate (*n* = 6), except for all Western blotting experiments, which were performed in quadruplicate (*n* = 4). ** for 0.01 > *p*-value > 0.001; *** for 0.001 > *p*-value.

In order to investigate the reasons for the improvement in egg quality, including an increase in albumen height and Haugh units, ovalbumin expression was examined. Ovalbumin is a protein found in the albumen, which is the clear liquid inside an egg. Ovalbumin is an important component of albumen. The magnum of the oviduct is the main site for ovalbumin protein formation. Therefore, we investigated the expression of ovalbumin and the morphological structure in the magnum. The results showed that both the NB205 and NBMK308 groups had significantly higher ovalbumin mRNA expression than the control group, as shown in Figure 2a. The NB205 and NBMK308 groups had significantly higher ovalbumin protein expression than the control group, with the NBMK308 group tending to be significantly higher than the NB205 group, as shown in Figure 2b. After staining with H&E and observation under the microscope, it was observed that the morphologies were evenly arranged in the magnum parts of the NB205 and NBMK308 groups (Figure 2c). The length of the primary and secondary folds was measured and it was found that there was a significant increase in the NB205 and NBMK308 groups (Figure 2d).

### 3.4. Regulation of Tight Junction (TJ) Proteins in the Magnum of Aging Laying Hens

**Figure 3:**
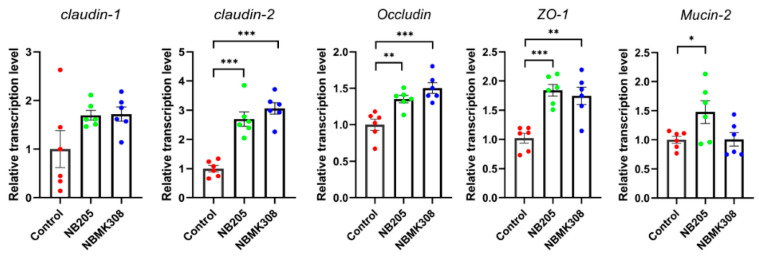
Regulation of TJ proteins in the magnum of aging laying hens. Expression of *claudin-1*, *claudin-2*, *occludin*, *mucin-2*, *ZO* (zonula occludens)*-1*, *FABP* (fatty acid binding protein)*-2* mRNA in the magnum quantified by qRT-PCR. All qRT-PCR experiments were performed in sextuplicate (*n* = 6). * for 0.05 > *p*-value > 0.01, ** for 0.01 > *p*-value > 0.001, and *** for 0.001 > *p*-value.

The results showed that in aging laying hens, the mRNA expression levels of TJ-related proteins such as *claudin-2*, *occludin*, *ZO-1,* and *FABP-2* were significantly higher in the NB205 and NBMK30 groups than in the control group. The gene *mucin-2* was significantly upregulated only in the NB205 group (Figure 3).

### 3.5. Regulation of Inflammation-Related Genes in Aging Laying Hens

**Figure 4:**
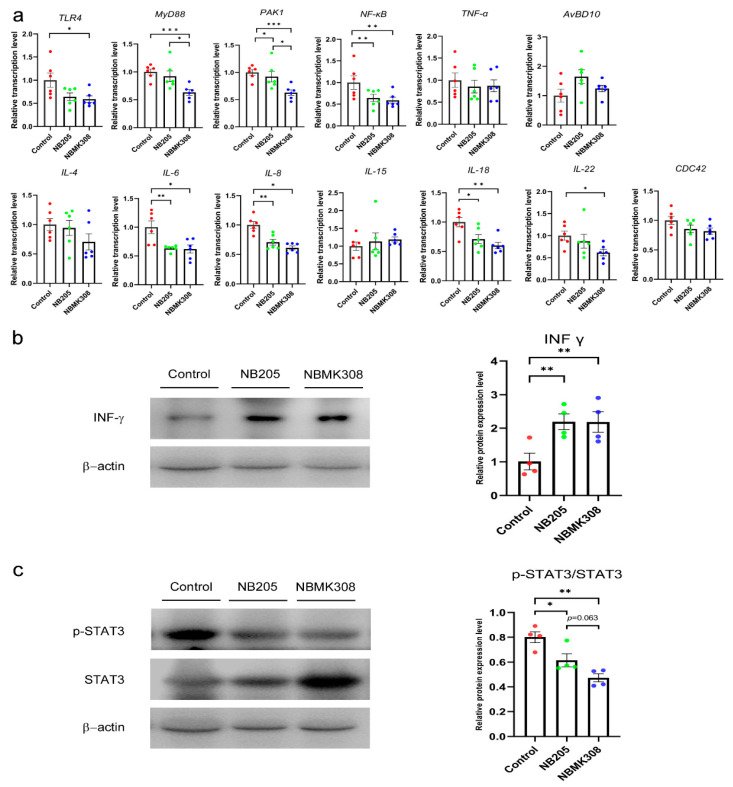
Regulation of inflammation-related genes in aging laying hens. (**a**) The mRNA expression levels of *TLR4*, *MyD88*, *PAK1*, *NF-κB*, *TNF-α*, *IL-4*, *IL-6*, *IL-8*, *IL-15*, *IL-18*, *IL-22*, *AvBD10,* and *CDC42* in magnum. TLR4, toll-like receptor 4; MyD88, myeloid differentiation factor 88; PAK1, p21-activated serine threonine kinase 1; NF-κB, nuclear factor kappa B; TNF-α, tumor necrosis factor-α; IL-4, interleukin-4; IL-6, interleukin-6; IL-8, interleukin-8; IL-15, interleukin-15; IL-18, interleukin-18; IL-22, interleukin-22; AvBD10, avian beta-defensin 10; CDC42, cell division cycle 42 protein. (**b**). Immune-related protein expression of INF-γ. (**c**). Immune-related protein expression of p-STAT3 and STAT3. * for 0.05 > *p*-value > 0.01, ** for 0.01 > *p*-value > 0.001, and *** for 0.001 > *p*-value.

In the experimental NB205 and NBMK308 groups, the relative mRNA expression levels of *IL-6*, *IL-8*, *IL-18*, *NF-κB,* and *PAK1* in the magnum of aging laying hens were significantly reduced. The relative mRNA expression levels of *IL-22* and *TLR4* were also significantly reduced in the NBMK308 group compared to the control group. The relative mRNA expression levels of *MyD88* and *PAK1* in the NBMK308 experimental group’s magnum were significantly lower than in the NB205 group (see Figure 4a). There was a significant increase in the expression of the immune-related protein INF-γ in both the NB205 and NBMK308 groups compared to the control group (Figure 4b). The NB205 and NBMK308 groups showed a significant decrease in p-STAT3/STAT3 protein expression, while there was a decreasing trend of p-STAT3/STAT3 expression in the NBMK308 group compared to the NB205 group (Figure 4c).

### 3.6. Regulation of Apoptosis-Related Genes in the Magnum of Aging Laying Hens

**Figure 5:**
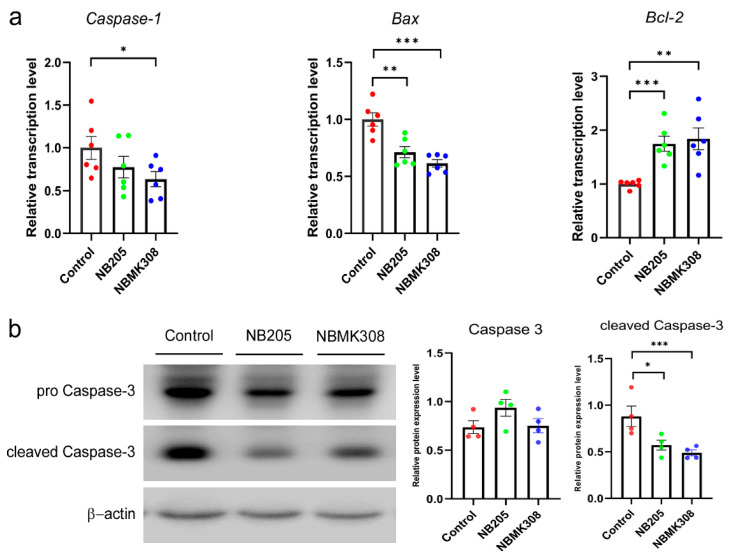
Regulation of apoptosis-related genes in the magnum of aging laying hens. (**a**) The mRNA expression levels of *caspase-1*, *Bax*, *Bcl-2* in the magnum. (**b**). Apoptosis-related protein expression of caspase-3 and cleaved caspase-3. * for 0.05 > *p*-value > 0.01, ** for 0.01 > *p*-value > 0.001, and *** for 0.001 > *p*-value.

Both the NB205 and NBMK308 groups showed a decrease in the expression of the *Bax* and an increase in the expression of the *Bcl-2* compared to the control group. Only the NBMK308 group showed a decrease in the expression of the *caspase-1* (Figure 5a). The protein expression of caspase-3 and cleaved caspase-3 showed a decreasing trend in both the NB205 and NBMK308 groups (Figure 5b).

### 3.7. Regulation of VKDPs-Related Genes in the Magnum of Aging Laying Hens

**Figure 6:**
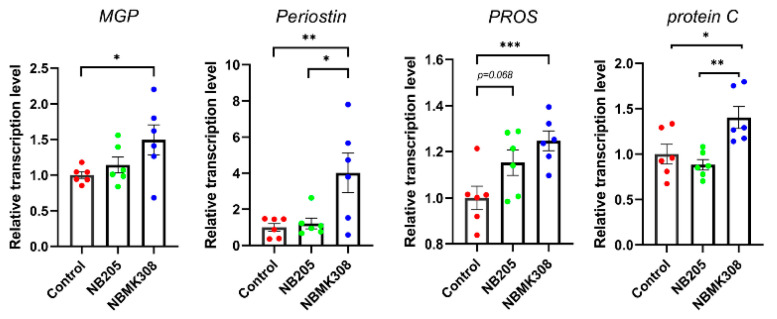
The mRNA expression levels of VKDPs such as *MGP*, *periostin*, *PROS,* and *protein C* in the magnum. * for 0.05 > *p*-value > 0.01, ** for 0.01 > *p*-value > 0.001, and *** for 0.001 > *p*-value.

The genes associated with VKDPs, such as *MGP*, *periostin*, *PROS,* and *protein C*, were upregulated in the NBMK308 group, while *PROS* showed an upward trend in the NB205 group (Figure 6).

## 4. Discussion

In recent years, several studies have investigated the effects of dietary supplementation with *B. subtilis* on reproductive performance and egg quality in poultry. *B. subtilis* can improve reproductive performance, egg quality, nutrient digestibility, and intestinal morphology in poultry. *B. subtilis* can improve serum antioxidant capacity, suggesting its potential as a dietary antioxidant for poultry. Taken together, these findings suggest that *B. subtilis* may be a promising dietary supplement to improve the poultry productivity and health [32,33,34,35,36]. *B. subtilis* may be a promising dietary supplement for improving productivity and health in poultry. *B. subtilis* natto is a subspecies or strain of *B. subtilis*. However, there are relatively few studies reporting the use of *B. subtilis* natto in aging laying hens. In this study, 81-week-old Hy-Line brown laying hens were fed a diet supplemented with either 0 or 1 × 10^8^ CFU/g of NB205 and NBMK308 for 8 weeks. We found that hens in the *B. subtilis* natto group had significantly higher albumen quality, including albumen height and Haugh unit, compared to the control group. The eggshell thickness of the *B. subtilis* natto group was significantly greater than that of the control group. The results of studies on the effects of dietary supplementation with *B. subtilis* natto on egg white quality and eggshell thickness in aging laying hens were promising. However, the mechanism by which *B. subtilis* natto improves these factors is not fully understood.

To investigate the reason of improvement in egg quality, particularly protein quality, in this study we focused on the magnum of the oviduct of aging laying hens to elucidate the effect of *B. subtilis* natto. The magnum part of the oviduct is a critical site for the synthesis of egg white protein, which is an essential component of egg quality [37]. There was a positive correlation between magnum morphology and albumen quality in Hy-Line Brown hens during the late laying period. Specifically, hens with larger magnums tended to produce eggs with higher albumen quality. Furthermore, dietary supplementation with tea polyphenols improved both magnum morphology and albumen quality, suggesting that the beneficial effects of tea polyphenols on albumen quality may be mediated, at least in part, by their effects on magnum morphology [38]. The magnum had the highest number of differentially expressed genes (DEGs) associated with egg quality, including genes involved in eggshell formation, calcium transport, and protein synthesis [39]. These findings highlight the importance of the magnum in determining egg quality and suggest that dietary interventions aimed at improving magnum function may be a promising strategy to improve egg quality in commercial egg production.

The magnum is a critical part of the chicken’s reproductive system responsible for the production of egg white, or albumen, which consists primarily of ovalbumin. Ovalbumin is a highly abundant protein and is the major component of egg white, accounting for up to 60% of the total protein content. The two species differ in the regulation of gene expression and protein synthesis, including ovalbumin, in magnum, which may contribute to the variation in egg quality and composition [40]. Zhao et al. (2016) found that ovalbumin expression in the oviductal magnum was positively correlated with egg laying rate [41]. In this study, the expression of ovalbumin mRNA and protein in the magnum were significantly increased by adding *B. subtilis* natto to the diet. The NB205 and NBMK308 groups showed significantly higher expression levels than the control group. A trend towards higher expression levels was observed in the NBMK308 group compared to the NB205 group. These results suggested that the addition of *B. subtilis* natto to the diet can regulate gene expression and protein synthesis, particularly ovalbumin, in the magnum and may play a role in determining egg quality and composition.

The magnum is responsible for the production of egg white and histological studies provide valuable insights into its structure and function. Histological studies have been crucial in understanding the structure and function of the magnum. The histology of the magnum is characterized by primary and secondary folds. The length of the primary folds increases during sexual maturation, which may increase the surface area available for protein secretion [42]. Primary folds are formed by mucosal ridges that run the length of the magnum, and their length can be an important indicator of magnum function. Secondary folds are formed by smaller mucosal ridges that branch off from the primary folds and form a more complex structure within the magnum. In this study, we found that NB205 and NBMK308 significantly increased the length of the primary and secondary folds in the magnum part of the oviduct. Primary folds play an important role in the secretion of egg white, which is the primary function of the magnum. The length of the primary folds is positively correlated with the secretory capacity of the magnum, meaning that a longer primary fold length may allow a greater volume of protein to be secreted [43]. An increase in secondary fold length may allow for a greater surface area for nutrient exchange between the magnum and the developing embryo. This may improve nutrient delivery to the embryo during early development, leading to improved embryo growth and development [44]. In conclusion, our study demonstrated that NB205 and NBMK308 can enhance the secretory capacity of the magnum and improve nutrient exchange between the magnum and the developing embryo, ultimately leading to improved embryonic growth and development.

The formation of the egg white requires strict regulation of the epithelial cells lining the oviduct, which is achieved by the formation of TJ between the cells. TJ proteins are essential components of the mucosal immune defense system that regulate the intercellular spaces between epithelial cells to strengthen the mucosal barrier, preventing the invasion of pathogens into submucosal tissues [45]. The actin cytoskeleton plays an important role in regulating epithelial permeability by controlling TJ proteins [46]. Deoxynivalenol impairs hepatic and intestinal gene expression of selected oxidative stressin broiler chickens, but the addition of an adsorbent shifts the effects to the distal parts of the small intestine [47]. Enteric pathogens and their toxins can disrupt the intestinal barrier by altering the TJ in chickens [48]. Elhamouly et al. (2019) investigated age-related changes in the innate immune defense system of the isthmic and uterine mucosa in laying hens [49]. The researchers found that the expression of TJ proteins, such as claudin-1 and occludin, was higher in the isthmic mucosa of young hens compared to older hens. In this study, it was found that the mRNA expression of TJ-related proteins was significantly upregulated in the NB205 and NBMK30 groups for genes such as *claudin-2*, *occludin*, *ZO-1,* and *FABP-2* in the magnum of aging laying hens compared to the control group. The gene mucin-2 was significantly upregulated only in the NB205 group. These findings highlight the critical role of TJ proteins in maintaining intestinal barrier function and provide insight into their regulation and potential implications for animal health. The results of this study suggested that supplementation of the NB205 and NBMK30 groups may have a beneficial effect on the regulation of TJ proteins in the mammary gland of aging laying hens, which could improve the integrity of the epithelial barrier and potentially reduce the risk of pathogen invasion. The upregulation of *claudin-2*, *occludin*, *ZO-1,* and *FABP-2* mRNA expression may indicate increased barrier function of the epithelial cells lining the magnum, while the upregulation of *mucin-2* mRNA expression in the NB205 group may indicate increased mucus production in the magnum. However, further studies are needed to confirm the efficacy of supplementation and to investigate its potential impact on egg quality and production.

The oviduct is an important reproductive organ in birds, responsible for the formation and transport of eggs. Inflammatory processes can affect the function of the oviduct and ultimately egg production. Aging is an inevitable process that affects many systems in the body, including the immune system. In laying hens, aging can lead to reduced egg production and egg quality due to chronic inflammation. Inflammation is a natural response to tissue injury or infection and involves the release of pro-inflammatory cytokines, chemokines, and other immune mediators [50]. However, chronic inflammation can contribute to the development of age-related diseases and impair immune function. These findings suggest that aging may contribute to chronic inflammation in the oviduct of laying hens. Inflammation is a common feature of aging and has been implicated in the development and progression of many age-related diseases. In this study, the mRNA levels of the pro-inflammatory cytokines *IL-6*, *IL-8*, *IL-18* and the inflammation-related genes *NF-κB* and *PAK1* were significantly lower in the oviduct of aging laying hens in the NB205 and NBMK308 groups. The mRNA levels of the pro-inflammatory cytokines *IL22* and *TLR4* were significantly reduced in the NBMK308 group, as were the mRNA levels of *MyD88* and *PAK1* compared to the NB205 group. There was a significant decrease in the expression of the immune-related protein p-STAT3/STAT3 in the magnum compared to the control group. There was a significant increase in INF-γ expression in the NBMK308 group, while there was a decreasing trend in p-STAT3/STAT3 expression in the NBMK308 group compared to the NB205 group.

The downregulation of pro-inflammatory cytokines in aging laying hens can benefit the uterine mucosa by reducing chronic inflammation associated with aging [51]. In aging, the downregulation of NF-κB can help reduce inflammation and improve metabolic function [52]. PAK1 is involved in cellular processes such as cell survival, proliferation, and differentiation, PAK1 is associated with age-related diseases, and downregulating PAK1 may have potential benefits in the aging process [53]. Downregulating TLR4 through PUM1 can help reduce cellular aging and osteoarthritis, indicating its potential therapeutic implications for age-related diseases [54]. Decreased p-STAT3/STAT3 levels may contribute to reduced inflammation and increased autophagy, which are important processes for maintaining healthy brain function [55]. In aging, increased levels of IFN-γ can have beneficial effects on the immune system, including promoting T cell activation and enhancing the function of macrophages [56]. The NB205 and NBMK308 groups showed significantly lower levels of pro-inflammatory cytokines and inflammation-related genes in the magnum of aging laying hens. Moreover, downregulation of NF-κB, TLR4, p-STAT3/STAT3, IFN-γ, and PAK1 may help reduce chronic inflammation, improve metabolic function, and alleviate cellular aging, respectively.

Several studies have focused on the effects of apoptosis and aging on the oviduct of laying hens and explored potential interventions to improve reproductive performance. Growth hormone [57], the mTOR signaling pathway [58], antioxidants [7,59], metformin [60], and rutin [61] have been used to modulate apoptosis in the oviduct. Apoptosis is a critical process in maintaining oviduct homeostasis in laying hens, and various factors and viral infections can modulate this process. Apoptosis, also known as programmed cell death, is a highly regulated process that plays a crucial role in maintaining tissue homeostasis and eliminating damaged or unwanted cells. In the oviduct, apoptosis is involved in several physiological processes, including cell proliferation, differentiation, and regression. Understanding the molecular mechanisms underlying these modulations may help to improve the health and productivity of laying hens.

Compared to the control group, both the NB205 and NBMK308 groups downregulated the expression of the *Bax* and upregulated the expression of the *Bcl-2*. The NBMK308 group alone downregulated the expression of the gene caspase-1, while the protein expression of cleaved caspase-3 showed a decreasing trend in both the NB205 and NBMK308 groups. The downregulation of Bax expression is associated with reduced oxidative damage and improved yolk precursor synthesis in aging laying hens [62]. FVS supplementation in aging laying hens upregulates the *Bcl-2* gene, promoting egg production and ovarian health by improving antioxidant capacity and hormone levels [62]. Downregulation of *caspase-1* expression during aging is associated with reduced chronic inflammation and improved insulin sensitivity [63]. Downregulation of caspase-3 during aging can help maintain the structural integrity of muscle tissue and improve meat quality [14,64]. Cleaved caspase-3 is a crucial protein in apoptosis, formed from the cleavage of caspase-3 during this process. This activation enables it to carry out its apoptotic function, leading to DNA fragmentation and cellular structure breakdown. As a widely used apoptosis marker, its presence indicates that the apoptotic pathway has been activated and the cell is undergoing programmed cell death. NB205 and NBMK308 improve the health and productivity of aging laying hens by regulating key apoptosis-related genes in the magnum. Specifically, they downregulate the expression of *Bax* and upregulate the expression of *Bcl-2*. These results may lead to benefits such as improved yolk precursor synthesis, reduced incidence of fibroid tumors, and chronic inflammation. NBMK308 downregulates the expression of *caspase-1*, further improving health outcomes during aging.

Compared to other common *B. subtilis* strains such as *B. subtilis* 168, NB205, and NBMK308 have a higher production of MK-7 after fermentation. In the study where the two strains were fed to aging laying hens, it was found that the NBMK308 group had significantly higher levels of MK-7 in their jejunal chyme than the NB205 group, and both groups had higher levels than the control group, demonstrating that the strains had colonized the gut and were secreting their metabolites. The genes associated with VKDPs such as MGP, periostin, PROS, and protein C were upregulated in the distended part of the intestine in the NBMK308 group, while PROS showed an upward trend in the NB205 group. MK-7, a type of VK, has a longer half-life than other forms of VK. VK is essential for blood clotting, bone metabolism, and cardiovascular health. In laying hens, VK has been shown to have beneficial effects on egg shell quality and bone health. VK supplementation can also alleviate bone calcium loss caused by Salmonella Enteritidis in laying hens through carboxylation of osteocalcin [65]. VKDPs have been shown to have numerous benefits in aging and age-related diseases. VKDPs promote bone health by facilitating the carboxylation of osteocalcin, which promotes calcium deposition in bone tissue; VKDPs also have anti-inflammatory effects by regulating the expression of genes involved in inflammation [66]. VKDPs have been shown to reduce the risk of cardiovascular disease by inhibiting the calcification of arterial walls [67]. Although there are differences in the expression of VKDPs between the NB205 and NBMK308 groups, they did not show differences in improving egg quality. Therefore, future research is planned to compare the differences in other indicators, such as gut and bone health, between these two strains when fed to aging laying hens to further validate the correlation between these indicators and VKDPs.

In summary, the study showed that supplementing the diets of aging laying hens with *B. subtilis* natto NB205 and its mutant NBMK308 led to an improvement in egg quality, including higher levels of albumen quality and eggshell thickness. Supplementation had a positive effect on the regulation of TJ proteins in the magnum part of oviduct, which could potentially reduce the risk of pathogen invasion and maintain normal albumin secretion. The study also found that supplementation reduced levels of pro-inflammatory cytokines and inflammation-related genes and improved the health and productivity of aging laying hens by regulating key apoptosis-related genes in the magnum. Notably, there were differences in the expression of VKDPs in the magnum between NB205 and NBMK308, but no significant differences in the improvement of egg quality. Further research is planned to investigate other indicators such as gut and bone health in aging laying hens to validate the correlation between these indicators and VKDPs. This study also showed that mutant bacteria had no adverse effects when fed to aging laying hens.

## 5. Conclusions

Supplementing aging laying hens’ diet with NB205 and NBMK308 improved egg quality by regulating ovalbumin expression, TJ proteins, pro-inflammatory cytokine levels, and key apoptosis-related genes in the magnum part of the oviduct. This study highlights the potential benefits of using *B. subtilis* natto in poultry production and provides a basis for future research in this area.

## Figures and Tables

**Table 1:** Composition and nutrient level of the basal diet.

Items	Content, g/kg	Nutrient Level	
Corn	638	ME Mcal/kg	2.65
Soybean meal	231	Crude protein, g/kg	150
Soybean oil	5	Methionine, g/kg	3.6
Granular stone meal	110	Calcium, g/kg	40
50% choline chloride	1.5	Available phosphorus, g/kg	1.2
Sodium chloride	3	Total phosphorus, g/kg	3.2
Zeolite meal	5.78		
methionine	1.5		
Phytase (10,000)	0.4		
Rice husk meal	2		
Sandoquine	0.02		
Vitamin premix ^a^	1.5		
Mineral premix ^b^	0.3		

^a^ Provided per kilogram of diet: vitamin A, 8000 IU; vitamin D_3_, 2400 IU; vitamin E, 40 IU; vitamin K3, 2 mg; vitamin B1, 2 mg; vitamin B2, 6.4 mg; vitamin B6, 3 mg; vitamin B12, 0.02 mg; folic acid, 1 mg; niacin, 30 mg; Capantothenate acid, 10 mg. ^b^ Provided per kilogram of diet: Cu, 8 mg; Zn, 80 mg; Fe, 60 mg; Mn, 80 mg; Se, 0.15 mg; I, 0.35 mg.

**Table 2:** Effects of supplementation with NB205 and NBMK308 on performance in aging laying hens.

Items (Performance)	Group			*p*-Value
	Control	NB205	NBMK308	
early stage 23 days				
ADFI ^a^	140.37 ± 2.026	141.01 ± 1.531	141.25 ± 1.464	0.732
Egg weight (g)	62.53 ± 0.360	62.03 ± 0.383	62.16 ± 0.613	0.486
Laying rate (%)	83.80 ± 3.100	84.35 ± 3.455	85.87 ± 2.66	0.643
FCR ^b^	2.70 ± 0.076	2.72 ± 0.091	2.66 ± 0.083	0.622
Ratio of unqualified and broken eggs (%)	1.52 ± 0.483	1.68 ± 0.634	2.42 ± 0.865	0.390
late stage 15 days				
ADFI ^a^	134.95 ± 2.758	140.03 ± 2.248	141.31 ± 1.774	0.077
Egg weight (g)	65.27 ± 0.528	65.00 ± 0.567	65.52 ± 0.720	0.574
Laying rate (%)	74.25 ± 4.582	76.42 ± 3.699	75.75 ± 2.295	0.696
FCR ^b^	2.87 ± 0.202	2.86 ± 0.139	2.86 ± 0.078	0.969
Ratio of unqualified and broken eggs (%)	2.94 ± 1.072	3.61 ± 1.044	4.84 ± 1.131	0.255

^a^ ADFI average daily feed intake, FCR ^b^ feed conversion rate. Experiments were performed in sextuplicate (*n* = 6).

**Table 3:** Effects of supplementation with NB205 and NBMK308 on egg quality in aging laying hens.

Items (Egg Quality)	Group			*p*-Value
	Control	NB205	NBMK308	
pre-experiment				
Yolk color	5.35 ± 0.160	5.40 ± 0.110	5.10 ± 0.110	0.411
Eggshell strength (kg/cm^3^)	3.40 ± 0.102	3.20 ± 0.121	3.25 ± 0.097	0.574
Haugh unit	80.85 ± 1.392	76.27 ± 2.166	81.12 ± 1.739	0.274
Albumen height (mm)	6.82 ± 0.204	6.27 ± 0.202	6.93 ± 0.223	0.212
Eggshell thickness (mm)	0.343 ± 0.004	0.340 ± 0.005	0.337 ± 0.003	0.748
3 weeks				
Yolk color	7.46 ± 0.150 ^a^	7.95 ± 0.080 ^b^	7.70 ± 0.110 ^ab^	0.027
Eggshell strength (kg/cm^3^)	3.20 ± 0.087	3.240 ± 0.065	3.05 ± 0.072	0.184
Haugh unit	81.44 ± 1.291	78.99 ± 0.816	80.53 ± 0.833	0.241
Albumen height (mm)	6.92 ± 0.155 ^a^	6.48 ± 0.101 ^ab^	6.80 ± 0.143 ^b^	0.079
Eggshell thickness (mm)	0.366 ± 0.004 ^a^	0.365 ± 0.003 ^ab^	0.389 ± 0.003 ^b^	<0.001
6 weeks				
Yolk color	8.08 ± 0.160	7.73 ± 0.19	7.53 ± 0.181	0.155
Eggshell strength (kg/cm^3^)	2.90 ± 0.075	3.05 ± 0.097	2.86 ± 0.092	0.275
Haugh unit	69.61 ± 1.603 ^a^	76.67 ± 1.261 ^b^	77.92 ± 1.210 ^b^	0.001
Albumen height (mm)	5.30 ± 0.160 ^a^	6.29 ± 0.146 ^b^	6.26 ± 0.190 ^b^	0.001
Eggshell thickness (mm)	0.375 ± 0.003 ^a^	0.396 ± 0.003 ^b^	0.390 ± 0.002 ^b^	<0.001

Experiments were performed in sextuplicate (*n* = 6). The superscript letters ‘a’ and ‘b’ in Table 3 represented significant differences between groups using the letter-based significance marking method. If two groups shared the same letter, it indicated that the difference between them was not significant, whereas if they had no letters in common, the difference was considered significant.

## Data Availability

The authors confirm that the data supporting the findings of this study are available within the article and its Supplementary Materials.

## Supplementary Materials

The following supporting information can be downloaded at: https://www.mdpi.com/article/10.3390/life13051109/s1, Table S1. Primers used in this study for qRT-PCR, F: forward, R: reverse. Figure S1. Calibration curves used for MK-7 quantification for Figure 1b ([28]).
