# Supplementary material for: Effects of Bacillus subtilis Natto NB205 and Its Mutant NBMK308 on Egg Quality in Aging Laying Hens
Source: Life (Basel). 2023 Apr 29;13(5):1109. doi: 10.3390/life13051109 (PMC10223476; doi:10.3390/life13051109)
Supplement: Supplementary file 1 [file life-13-01109-s001.zip › life-2305549-supplementary.pdf]

Supplementary materials for

Effects of *Bacillus subtilis* natto NB205 and Its Mutant NBMK308 on Egg Quality in Aging Laying Hens.

Chaoyong Liao, Jian Cui, Jiaqi Lei, Yuming Guo and Bingkun Zhang *

State Key Laboratory of Animal Nutrition, Department of Animal Nutrition and Feed Science, College of Animal Science and Technology, China Agricultural University, Beijing 100193, China

* Correspondence: Correspondence: bingkunzhang@126.com

Table S1. Primers used in this study for qRT-PCR, F: forward, R: reverse.

Primer	Sequence (5'-3')
β-actin F	CAACACAGTGCTGTCTGGTGGTAC
β-actin R	CTCCTGCTTGCTGATCCACATCTG
Ovalbumin F	ttattcgttcagccttgc
Ovalbumin R	tttctcccacagtccttt
claudin-1 F	CATACTCCTGGGTCTGGTTGGT
claudin-1 R	GACAGCCATCCGCATCTTCT
occludin F	ACGGCAGCACCTACCTCAA
occludin R	GGGCGAAGAAGCAGATGAG
ZO-1 F	CTTCAGGTGTTTCTCTTCCTCCTC
ZO-1 R	CTGTGGTTTCATGGCTGGATC
Mucin-2 F	TTCATGATGCCTGCTCTTGTG
Mucin-2 R	CCTGAGCCTTGGTACATTCTTGT
TLR-4 F	CCACTATTCGGTTGGTGGAC
TLR-4 R	ACAGCTTCTCAGCAGGCAAT
MyD88 F	TGCAAGACCATGAAGAACGA
MyD88 R	TCACGGCAGCAAGAGAGATT
PAK1 F	GCTACAGGACAGGAGGTTGC
PAK1 R	ACGACATCTGTTAGGGAGCC
NF-κb F	TGGAGAAGGCTATGCAGCTT
NF-κb R	CATCCTGGACAGCAGTGAGA
TNF-α F	GAGCGTTGACTTGGCTGTC
TNF-α R	AAGCAACAACCAGCTATGCAC
AvBD10 F	TAAACTGCTGTGCCAAGATTCCG
AvBD10 R	AGCTCCTCAAGGCAGTGGAA
IL-4 F	GCTCTCAGTGCCGCTGATG
IL-4 R	GAAACCTCTCCCTGGATGTCAT
IL-6 F	CGCCCAGAAATCCCTCCTC
IL-6 R	AGGCACTGAAACTCCTGGTC
IL-8 F	ATGAACGGCAAGCTTGGAGCTG
IL-8 R	TCCAAGCACACCTCTCTTCCATCC
IL-15 F	TGGAGCTGATCAAGACATCTG
IL-15 R	CATTACAGGTTCCTGGCATTC
IL-18 F	TGATGAGCTGGAATGCGATG
IL-18 R	TGATGAGCTGGAATGCGATG
IL-22 F	GGTTGTCTTCTGCTGTTGTTGCTG
IL-22 R	GCCAAGGTGTAGGTGCGATTCC
CDC42 F	AGCCCATAACTCCAGAGACG
CDC42 R	CATTCTTTAGGCCTTTCTGCGT
Caspase-1 F	GTGAGGCAGTACGGTGGTGTT
Caspase-1 R	TGTCCCCTTTGGGTTCCTGT

Bax F	ATCGTCGCCTTCTTCGAGTT
Bax R	ATCCCATCCTCCGTTGTCCT
Bcl-2 F	TCGCGCCGCTACCAGAGGGACTTC
Bcl-2 R	CCGGTTGACGCTCTCGACGCACAT
MGP F	GGCCAATGGCTTCATACGAG
MGP R	ATCTCCCTCTGACGTTCCTGA
Periostin F	GCAGGGAGCTGGAACTGAG
Periostin R	CTTGCTGATCCCCCTTGTGT
PROS F	TAGGTGGCCTGCCAGATGTTC
PROS R	CCAGCTGTCTGCTGTTGACC
protein C F	GTGTCTGATGGCAAGGGGAG
protein C R	CAGCAGAAGTCCCCAGCAG

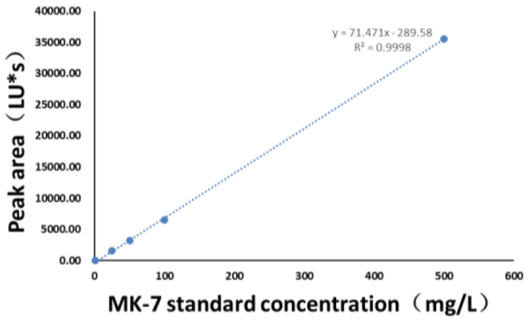

Figure S1. Calibration curves used for MK-7 quantification for Figure 1b ([28]).
